# BRD10 Regulates DZIP1 Localization at the Primary Cilium During Mitral Valvulogenesis

**DOI:** 10.3390/jcdd13100516

**Published:** 2026-10-14

**Authors:** Brian Loizzi, Lilong Guo, Matthew Huff, Prakrit Subba, Magdalena K. Brei, Ranan Phookan, Roman Fenner, Kathryn Byerly, Baylee Westbury, Sarah Dooley, Russell A. Norris

**Affiliations:** 1 Department of Regenerative Medicine and Cell Biology, Medical University of South Carolina, Charleston, SC 29407, USA; loizzi@musc.edu (B.L.); guol@musc.edu (L.G.); huffmat@musc.edu (M.H.); subba@musc.edu (P.S.); maddiebrei@gmail.com (M.K.B.); phookan@musc.edu (R.P.); fennerro@musc.edu (R.F.); byerlyk@musc.edu (K.B.); westburb@musc.edu (B.W.); dooleysarah1@gmail.com (S.D.); 2 Department of Pathology, Medical University of South Carolina, Charleston, SC 29407, USA; 3 Department of Neurosurgery, Medical University of South Carolina, Charleston, SC 29407, USA

**Keywords:** mitral valve prolapse, valvulogenesis, DZIP1, BRD10, primary cilia, ciliogenesis, bromodomain proteins, acetylated α-tubulin, basal body, cardiac development

## Abstract

Mitral valve prolapse (MVP) is a common congenital valve disorder that has been linked to defects in primary cilia during cardiac development, yet the mechanisms connecting ciliary regulation to valvulogenesis remain incompletely understood. As DZIP1 is required for ciliogenesis and has been shown to be involved in MVP pathogenesis, we sought to identify interacting partners that may regulate its localization and function. Using an unbiased yeast two-hybrid screen of a human heart cDNA library, we identified BRD10, a previously uncharacterized bromodomain-containing protein, as a DZIP1-interacting partner. Co-immunoprecipitation confirmed the DZIP1–BRD10 interaction and mapped the binding region to a conserved domain containing a putative nuclear localization sequence. RNAscope and immunofluorescence demonstrated that BRD10 and DZIP1 are co-expressed in developing murine valve cells, with BRD10 localizing near the basal body and transition zone of the primary cilium. BRD10 also interacted with acetylated α-tubulin, supporting a relationship with ciliary microtubule structures. Nuclear–cytoplasmic fractionation further showed that BRD10 promotes cytoplasmic enrichment of DZIP1. Together, these findings identify BRD10 as a novel regulator of DZIP1 subcellular localization and suggest that bromodomain-mediated recruitment at the primary cilium may contribute to normal valvulogenesis and developmental mechanisms relevant to MVP.

## 1. Introduction

Valvulogenesis is a coordinated developmental process that requires the spatial and temporal integration of interconnected signaling pathways to ensure proper cardiac valve form and function [1,2,3]. Disruption of these pathways can have lasting consequences for valve architecture and biomechanical integrity. Primary cilia have emerged as important regulators of endothelial-to-mesenchymal transition (EndoMT), cell migration, and the organization of mesenchymal cells within the developing endocardial cushions [4,5,6]. These cellular appendages are membrane-bound solitary protrusions that consist of a microtubule-based axoneme, a transition zone, and a basal body that anchors the cilium to the cell. Associated with the primary ciliary membrane are numerous receptors that enable cells to sense and integrate extracellular cues into molecular and cellular changes. By functioning as signaling hubs, these structures regulate downstream pathways that lead to establishment of proper extracellular matrix (ECM) distribution. Accordingly, disruption of ciliogenesis during cardiac valve development can alter ECM deposition and leaflet structure through expansion of the proteoglycan-rich spongiosa, a defining feature of myxomatous valve degeneration and mitral valve prolapse (MVP) [7,8,9,10,11].

Previous work from our group and others has demonstrated that loss-of-function mutations in the ciliary protein DZIP1 are causative for MVP in humans and can be linked to developmental errors in ciliogenesis [9,11]. Although many of the core proteins and trafficking pathways required for ciliogenesis have been heavily studied, the molecular mechanisms through which DZIP1 coordinates ciliary assembly and stability remain incompletely understood. To identify additional components of the DZIP1-dependent ciliary machinery, we performed an unbiased proteomic screen for DZIP1-interacting proteins and subsequently evaluated candidate partners for roles in normal ciliogenesis. Through these studies, we identified bromodomain-containing protein 10 (BRD10) as a previously unrecognized component of the primary cilia. These data establish a potential molecular link between BRD10, DZIP1-dependent ciliogenesis, and the developmental pathogenesis of MVP.

## 2. Materials and Methods

### 2.1. Yeast 2-Hybrid Screen

Yeast two-hybrid screens were performed by Hybrigenics Services (Paris, France; www.hybrigenics-services.com (accessed on 26 November 2025)) as previously described. The coding sequence of the full-length DZIP1 isoform 2 (NM_198968.4) was cloned in frame with the LexA DNA-binding domain (DBD) into plasmid pB27 on the C-terminus of Gal4 to generate a Gal4–bait fusion. This bait protein was used to screen a total of 113 million clones from a human adult and fetal heart cDNA library. In total, 247 prey fragments were identified and sequenced at their 5′ and 3′ junctions.

A Predicted Biological Score (PBS) was attributed to each interaction as previously described [9,12]. This score indicates the likelihood of an identified bait–prey interaction occurring. This score is based on a comparison of the number of prey fragments found for an interaction and the probability that this interaction would be found at random. The PBS is scored from A to F, with A being the highest confidence for an interaction and D being the lowest confidence. Scores of E and F represent likely non-specific interactions that either involve highly connected prey domains (E) or experimentally proven technical artifacts, such as LexA or Gal4 binders (F). A total of 80 clones with confidence scores of A (very high confidence) or B (high confidence) from the original 247 clones identified were prioritized for follow-up confirmation.

### 2.2. Plasmids and Vectors

Control human BRD10 plasmid was purchased from GenScript (Nanjing, China) (CloneID: OHu76991). Truncations of this plasmid were sub-cloned with a C-terminal FLAG epitope tag using the pTARGET Mammalian Expression Vector System (Promega, Madison, WI, USA, A1410). The control human DZIP1-Myc-DDK plasmid was purchased from Origene (Rockville, MD, USA, SKU: RC207188). The Myc-DDK epitopes were replaced with a HA-epitope tag at the C-terminus via the QuickChange II XL Site-Directed Mutagenesis Kit (Agilent Technologies, Santa Clara, CA, USA, 200521) based on the manufacturer’s recommendations. P2A bicistronic constructs were custom-generated by VectorBuilder (Chicago, IL, USA). These included 3xOLLAS-BRD10-3xV5/GFP-3xHA (Vector ID:VB251106-1064ktu), eGFP-3xV5/DZIP1-3xHA (Vector ID: VB251027-1531nna), 3xOLLAS-BRD10-3xV5/DZIP1-3xHA (Vector ID: VB251106-1063ssn), and eGFP-3xV5/mCherry-3xHA (Vector ID: VB251027-1530crt). Detailed information about each vector can be found at vectorbuilder.com (accessed 6 November 2025). All plasmids and vectors were sequenced via Genewiz (Morrisville, NC, USA) before use.

### 2.3. RNAscope

RNAscope^®^ Multiplex Fluorescent v2 Assay (ACD BioTechne, Minneapolis, MN, USA) User Manual UM323100) was performed on a minimum of 3 independent formalin-fixed, paraffin-embedded embryonic day 13.5 (E13.5) embryos, postnatal day 0 (P0) neonatal hearts and adult hearts from C57BL6/J mouse lines. Embryos and tissues were fixed in 10% neutral buffered formalin at room temperature for ~24 h and processed for paraffin embedding according to the manufacturer’s protocol. The user manual was followed for the RNAscope procedure. RNAscope probes for Mm-*Dzip1* (ACD BioTechne, 1252191-C2) and Mm-*Kiaa2026* (ACD BioTechne, 1252821-C1) were used. Slides were imaged using the Leica SP8 confocal microscope (Leica Microsystems, Inc., Deer Park, IL, USA).

### 2.4. Co-IP and Western Blotting

HEK293Ts were seeded at 5 × 10^5^ cells per well on a 6-well plate and cultured in DMEM (ThermoFisher, Waltham, MA, USA, 11320033) containing 10% fetal bovine serum (VWR, Radnor, PA, USA, 16777-014) and 1% pen-strep (VWR, 15140112). Cells were co-transfected 24 h later with DZIP1 and BRD10 plasmids using FuGENE HD transfection Reagent (Promega, E2311). The cells were then lysed 72 h later using ice-cold lysis buffer (25 mM Tris HCL, 150 mM NaCl, 1% NP-40, 5% glycerol) containing protease and phosphatase inhibitor (ThermoFisher, 78440, dilution 1:500). Lysates were incubated on ice for 30 min with intermittent vortexing. Cell lysates containing 400 μg of protein were precleared by adding 2.5 μL of IgG derived from the same species as the primary antibody and 25 μL of Pierce Protein A/G Agarose beads (ThermoFisher, 20421) and rotated for 2 h at 4 °C. Next, 5 μL of primary antibody or IgG with 50 μL of Pierce Protein A/G Agarose beads was added to pre-cleared cell lysates and incubated overnight at 4 °C. Beads were collected and washed 5 times with 1 mL of ice-cold lysis buffer prior to SDS-PAGE. For acetylated α-tubulin Co-IP, human cardiac fibroblasts (Cell Applications, San Diego, CA, USA, 306V-05a) were seeded at 3 × 10^5^ cells per well on a 6-well plate and cultured in HCF-specific culture medium (Cell Applications; 315–500) to reach confluence in 48 h. Cells were then trypsinized, and lysate was collected and processed for Co-IP as above. The primary antibodies used for Co-IP assays and Western blotting were as follows: mouse anti-Flag M2 (Sigma, F1804, dilution 1:1000), rabbit anti-HA (Invitrogen, Carlsbad, CA, USA, MA5-27915, dilution 1:1000), rabbit anti-V5 (Cell Signaling, Danvers, MA, USA, 13202S, dilution 1:1000), rabbit KIAA2026 (Biorbyt, Cambridge, UK, orb450648, dilution 1:1000, discontinued), rabbit DZIP1 (Proteintech, Rosemont, IL, USA, 13779-1-AP, dilution 1:1000). Anti-mouse (Sigma-Aldrich, St. Louis, MO, USA, A9044, dilution 1:10,000) and anti-rabbit (Sigma-Aldrich, A9169, dilution 1:10,000) horseradish peroxidase (HRP)-conjugated secondary antibodies were used.

### 2.5. BRD10 Peptide and Streptavidin Pull-Down Methods

BRD10 peptides were synthesized by Genescript with biotin at the carboxy-terminus. For peptide Co-IP, *Dzip1*-tGFP-transfected HEK293 cells were incubated with Pierce Streptavidin Magnetic Beads (ThermoFisher, 88816) and biotinylated peptides at 4 °C overnight. The magnetic beads were washed, and the complexes were eluted according to the manufacturer’s recommendation prior to SDS-PAGE. Primary antibodies used for Co-IP assays and Western blotting were as follows: α-chicken GFP (Aves Lab, Davis, CA, USA, GFP-1020, dilution 1:1000), and horseradish peroxidase (HRP)-conjugated secondary antibodies (Sigma-Aldrich, AP162P, dilution 1:10,000). All Co-IP and Western blot analyses were repeated a minimum of three times in independent biological replicates.

### 2.6. Immunohistochemistry

Immunohistochemical and fluorescence stains were performed on 5 μm, formalin-fixed, paraffin-embedded sections from embryonic timepoint E13.5 (*n* = 3). Sections were deparaffinized and rehydrated through xylene and graded alcohols before antigen unmasking in 0.93% citric acid (H-3300; Vector Laboratories, Burlingame, CA, USA) and high-pressure cooking for 1 min. Sections were then treated for 1 h at room temperature with a blocking buffer of PBS (Sigma, St. Louis, MO, USA) containing 1% BSA (Sigma, B4287-25G) followed by overnight probing of primary antibodies. The following day, sections were washed with PBS and treated for 1 h at room temperature with secondary antibodies and counter stained with Hoechst 33258 (Invitrogen, H3569, dilution 1:10,000). Slides were cover slipped with SlowFade Gold (Invitrogen, S36936). The primary antibodies used were: mouse gamma-tubulin (Sigma, T6557, dilution 1:100), mouse acetylated α-tubulin (Sigma, T6793, 1:100), and rabbit BRD10 (Atlas Antibodies, Stockholm, Sweden, HPA002109, dilution 1:100). Images of immunostained sections were captured with the ECHO Revolution Confocal (Discover Echo Inc., San Diego, CA, USA) or Leica TCS SP8 AOBS Confocal Microscope System (Leica Microsystems, Inc., Exton, PA, USA). Z-stacks were set by finding the highest and lowest depth with visible fluorescence and using optimized system settings to determine steps. The Z-stacks were then compiled to form maximum projection images or used to generate 3D reconstructions via Imaris.

### 2.7. Immunocytochemistry

NIH3T3 cells were seeded at 3 × 10^5^ cells per well in rat-tail collagen I (VWR, 47743-656)-coated 6-well plates containing a glass cover slip and cultured in DMEM (ThermoFisher, 11320033) containing 10% fetal bovine serum (VWR, 16777-014) and 1% pen-strep (VWR, 15140112); 24 h later, the medium was replaced with DMEM (ThermoFisher, 11320033) containing 1% pen-strep (VWR, 15140112) and cultured for another 24 h. Cells were removed from the incubator and placed on ice for 30 min. The medium was removed and replaced with ice-cold extraction buffer (20 mM HEPES (pH 7.4), 50 nM NaCl, 3 mM MgCl_2_, 300 mM sucrose, and 0.5% Triton-X 100) for 45 s. The extraction buffer was removed, and cells were fixed with ice-cold methanol for 10 min at −20 °C. The cells were washed with PBS and blocked in PBS containing 1% BSA (Sigma, B4287-25G) for 1 h at room temperature. Primary antibodies were then added and incubated at 4 °C overnight. The following day, cells were washed with PBS, and secondary antibodies were added. Cells were counterstained with Hoechst 33258 (Invitrogen, H3569, dilution 1:10,000) and mounted onto a glass slide with SlowFade Gold (Invitrogen, S36936). Primary antibodies used were mouse acetylated α-tubulin (Sigma, T6793, dilution 1:100) and rabbit BRD10 (Atlas Antibodies, HPA002109, dilution 1:100). Secondary antibodies used were goat anti-mouse Alexa Fluor 568 (Invitrogen, A11004, dilution 1:100) and goat anti-rabbit Cyanine5 (Invitrogen, A10523, dilution 1:100). Images of immunostained sections were captured with the ECHO Revolution Confocal (Discover Echo Inc., San Diego, CA, USA) or Leica TCS SP8 AOBS Confocal Microscope System (Leica Microsystems, Inc., Exton, PA, USA). Z-stacks were set by finding the highest and lowest depth with visible fluorescence and using optimized system settings to determine steps. All ICC experiments were performed in at least three independent biological replicates, with multiple cells imaged and analyzed from each replicate to confirm reproducibility of the observed staining patterns.

### 2.8. Nuclear and Cytoplasmic Protein Extraction

HEK293Ts were seeded at 5 × 10^5^ cells per well on a 6-well plate and cultured in DMEM (ThermoFisher, 11320033) containing 10% fetal bovine serum (VWR, 16777-014) and 1% pen-strep (VWR, 15140112). The cells were transfected 24 h later with previously described P2A constructs using Fugene 4K at a ratio of 2 μg DNA to 8 μL Fugene 4K reagent. GFP expression was confirmed 72 h post-transfection, and the cells were mechanically dissociated, and nuclear and cytoplasmic fragmentation was done following the protocol provided in the NE-PER Nuclear and Cytoplasmic Extraction Reagents Kit (ThermoFisher, 78835). Western blotting was then performed as described above. GAPDH (ProteinTech, 60004-1-Ig, dilution 1:1000) and Histone H4 (Cell Signaling, 2935s, dilution 1:1000) were used as cytoplasmic and nuclear markers, respectively. Statistical analyses were performed using GraphPad Prism (version 10, GraphPad Software, San Diego, CA, USA). Nuclear and cytoplasmic fractionation experiments were performed in four independent biological replicates. Western blot band intensities were quantified for each independent biological replicate using densitometry. For each nuclear and cytoplasmic fraction, the target protein signal was normalized to the total protein signal within the corresponding lane as determined by Ponceau S staining. Nuclear and cytoplasmic fractions were analyzed independently, and normalized DZIP1 abundance was compared between cells expressing DZIP1 alone and cells co-expressing DZIP1 and BRD10. BRD10 abundance was analyzed similarly in cells expressing BRD10 alone versus BRD10 together with DZIP1. In addition, the cytoplasmic-to-nuclear (C:N) ratio was calculated for each biological replicate using the normalized values from the corresponding fractions. Because nuclear and cytoplasmic fractions were processed and normalized independently, raw Western blot band intensity across fractions was not interpreted as a measure of total cellular protein abundance. Fractionation experiments were performed in four independent biological replicates (*n* = 4). Statistical analyses were performed using GraphPad Prism version 10, with significance defined as *p* < 0.05.

## 3. Results

**Figure 1:**
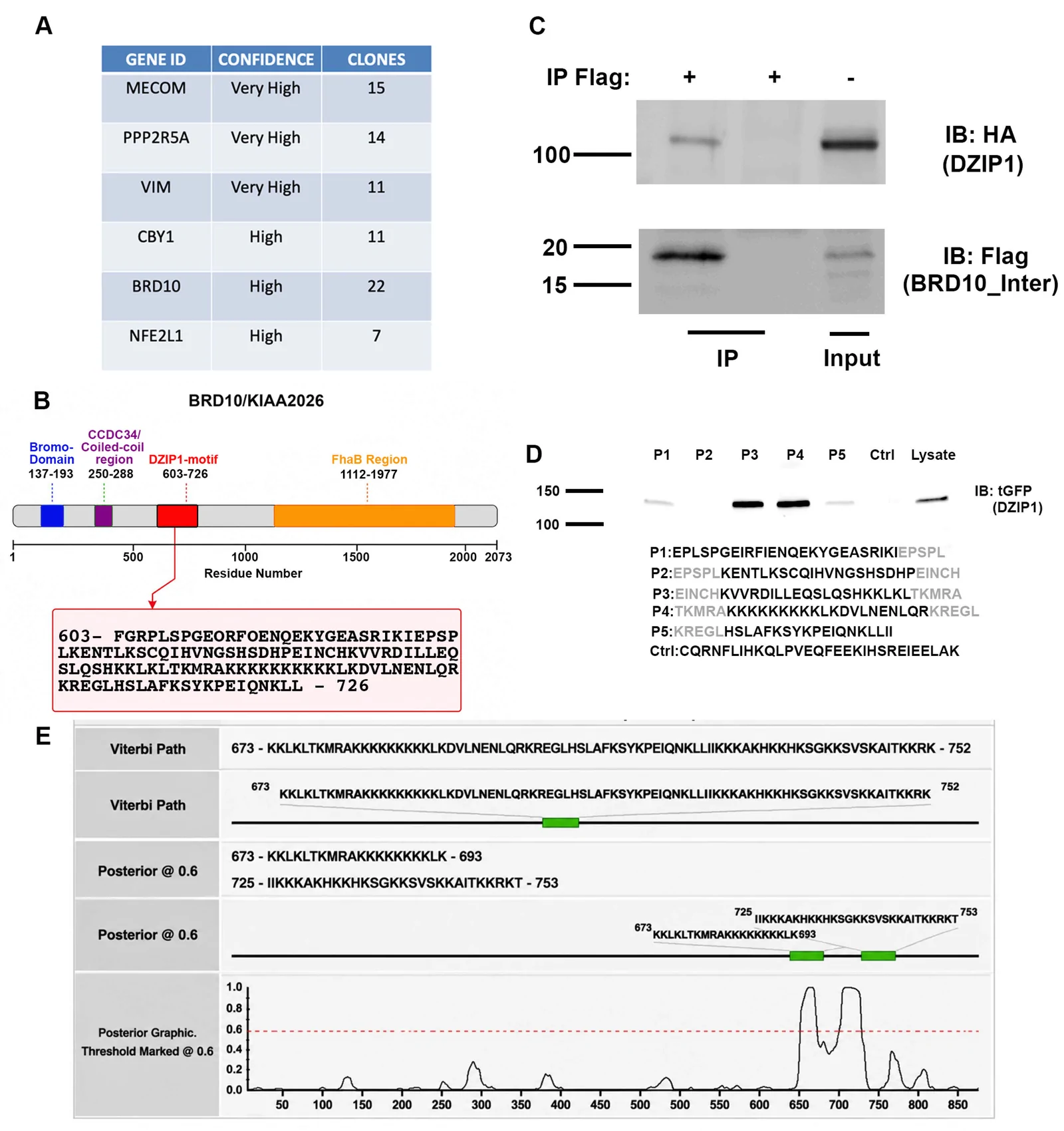
Identification and mapping of the BRD10–DZIP1 interaction. (**A**) The yeast two-hybrid screen identified several high-confidence interacting proteins, including BRD10. The table summarizes the top interactors, confidence scores, and clone numbers. (**B**) Schematic representation of BRD10 showing predicted protein domains and the region implicated in DZIP1 binding. The boxed sequence denotes the candidate DZIP1-interaction region. (**C**) Co-IP confirmed interaction between DZIP1 and the BRD10 interaction fragment. (**D**) Streptavidin pull-down assays identified the strongest interaction observed in peptides 3 and 4. (**E**) Sequence and computational analyses of BRD10 showing the upper “Viterbi Path” tracks, which show the predicted NLS calls from the sequence-based algorithm. The green segment marks amino-acid stretches predicted to function as nuclear localization sequences. The posterior probability plots underneath show the confidence of those predictions; the regions rising above the threshold line (red dotted line) correspond to the predicted NLSs. We identified two putative NLS regions at amino acids 673–693 and 725–753. The first predicted NLS overlaps the DZIP1-interacting region mapped in Figure 1B,D, including the P3/P4 peptide region.

A two-hybrid screen of a human heart cDNA library was performed with full-length DZIP1 as a bait to identify novel DZIP1-interacting partners. In total, 113 million clones were screened, resulting in the identification of 80 clones from six proteins (MECOM, PPP2R5A, VIM, CBY1, BRD10, and NFE2L1) with a score of A (very high) or B (high) indicating their probability of being interactors (Figure 1A). MECOM is a transcription factor known to be required for proper craniofacial and cochlear development through regulation of TGFβ, Wnt, and Notch signaling pathways [13,14]. PPP2R5A is a regulatory subunit of PP2A that has been implicated in arrhythmogenicity and tumor suppression [15,16]. PP2A has been shown to dephosphorylate DZIP1, affecting Gli protein turnover [17]. Vimentin has long been established as required for proper EndoMT due to its role in cytoskeletal remodeling and cell migration [18,19]. A mechanistic link between DZIP1 and vimentin has been established in oral squamous cell carcinoma, wherein DZIP1 overexpression increased vimentin expression presumably through the Gli1/3 signaling pathway [20]. Cby1 is known to be required for primary ciliogensis and has been shown to interact with DZIP1 and affect β-catenin signaling within the developing mitral valve [9,21]. Finally, NFE2L1 is an endoplasmic reticulum (ER) membrane sensor, which, during periods of ER stress, translocates to the nucleus and acts as a transcription factor for genes in various stress response pathways [22]. BRD10 (also known as KIAA2026) is an uncharacterized bromodomain-containing protein with unknown function within the cell. Broadly, bromodomain-containing proteins interact with acetylated lysine groups on various target proteins and have a wide variety of functions including epigenetic reading, histone modification, chromatin remodeling, and protein scaffolding [23,24,25,26]. Therefore, we focused on BRD10 (also known as KIAA2026) for further analysis because of its novelty, limited functional characterization, and strong representation in the screen, yielding the highest number of positive clones (22 total).

The high degree of overlap among independent clones enabled refinement of the putative DZIP1–BRD10 interaction motif (Figure 1B). DNA encoding this candidate BRD10 interaction region was subsequently tested for its ability to bind DZIP1 using co-transfection followed by co-immunoprecipitation (Co-IP). Western blot analyses confirmed the ability for the BRD10 fragment to interact with DZIP1 (Figure 1C). To further refine the interaction motif, we dissected the motif into five overlapping peptides tagged with C-terminal biotin moieties and repeated the Co-IP over streptavidin beads. A positive interaction was identified, with the strongest interaction based on Western blot intensity occurring in peptides 3 and 4 (Figure 1D). Sequence and computational analysis of full-length BRD10 identified a bromodomain-containing region (aa 137–193), a coiled-coil region (aa 250–288), a large FhaB-like region (aa 1112–1977), and two putative nuclear localization signals (NLSs), highlighted in green and indicated by arrowheads, spanning approximately aa 673–693 and aa 725–753 within the DZIP1-interacting domain of BRD10, indicated in red (Figure 1B,E). Notably, the first predicted NLS directly overlaps the DZIP1-binding region identified in our screen, raising the possibility that interactions between DZIP1 and BRD10 may impact their respective subcellular localizations.

**Figure 2:**
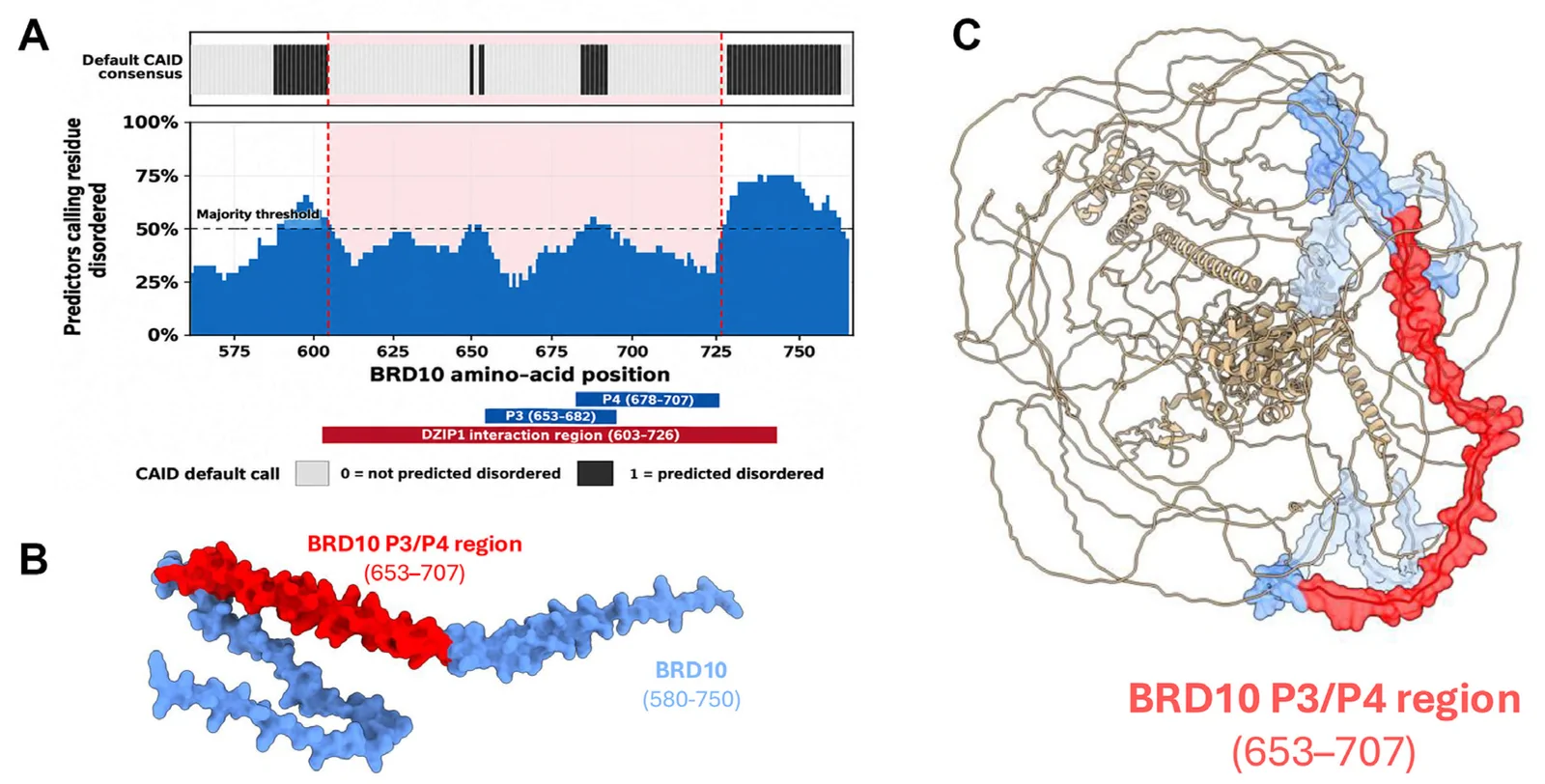
Structural prediction of the DZIP1-interaction region on BRD10. (**A**) Sequence-based disorder analysis of BRD10 showing predicted intrinsically disordered regions across the protein and the location of the experimentally defined DZIP1-interaction domain. The P3/P4 peptide region is indicated within the broader interaction segment. (**B**) AlphaFold-based structural model of BRD10 residues 580–750 highlighting the P3/P4 interaction region (red; aa 653–707), which lies within an extended and surface-accessible portion of the protein. (**C**) Predicted full-length BRD10 structure showing the position of the P3/P4 interaction region on the protein surface.

To further characterize the BRD10 region implicated in DZIP1 binding, we examined its predicted structural properties using sequence-based disorder analysis and AlphaFold modeling (Figure 2) [27,28]. The DZIP1-interaction region approximately spans amino acids 603–726 of BRD10, encompassing peptides P3 and P4, which showed the strongest DZIP1 binding in the peptide pull-down studies. Disorder prediction using CAID indicated that much of this region is predicted to be intrinsically disordered or conformationally flexible, with local variation in disorder probability across the interaction domain (Figure 2A). Notably, peptides P3 (aa 653–682) and P4 (aa 678–707) lie within this broader interaction region and overlap the portion of BRD10 previously identified as containing a putative nuclear localization sequence. AlphaFold-based structural visualization of BRD10 residues 580–750 further demonstrated that the P3/P4 interaction region occupies an extended, surface-accessible segment rather than being buried within a compact globular domain (Figure 2B). When mapped onto the predicted full-length BRD10 structure, the P3/P4 region remained prominently exposed on the protein surface (Figure 2C). This predicted accessibility is consistent with the ability of this region to participate in intermolecular interactions and supports the biochemical identification of P3 and P4 as likely contributors to DZIP1 binding. Together, these analyses suggest that DZIP1 interacts with a relatively flexible and accessible region of BRD10, providing a putative structural basis for the observed BRD10–DZIP1 association.

**Figure 3:**
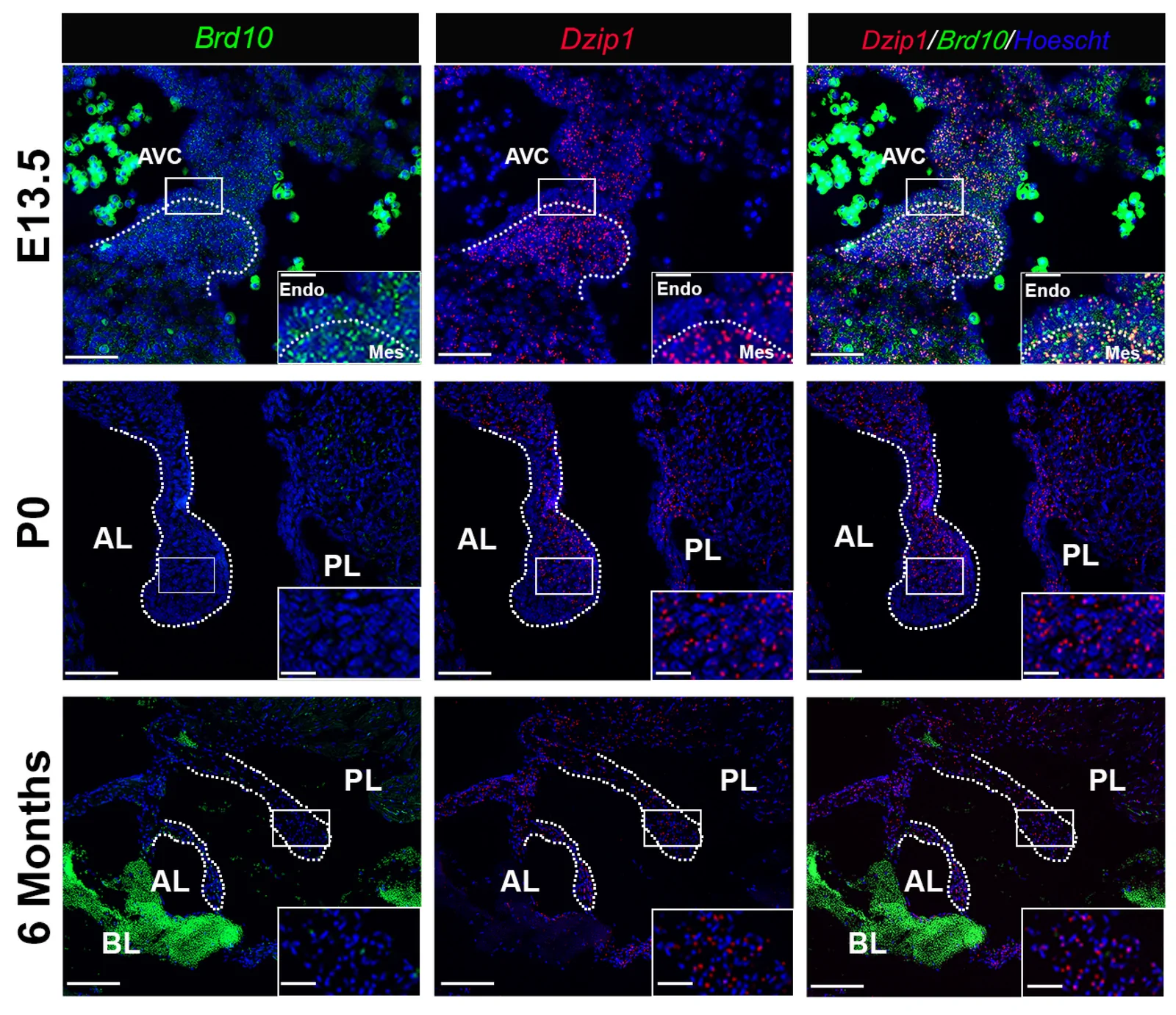
Developmental expression of *Brd10* and *Dzip1* in the mitral valve. RNAscope analysis of *Brd10* (green) and *Dzip1* (red) expression in murine atrioventricular cushions and mitral valves on embryonic day 13.5 (E13.5) and postnatal day 0 (P0) and at 6 months of age. At E13.5, *Brd10* is expressed in both endothelial (endo) and mesenchymal (mes) cells of the developing atrioventricular cushions, whereas *Dzip1* expression is predominantly mesenchymal. Co-expression is evident in cushion mesenchymal cells. At P0, *Dzip1* expression persists within the mitral valve mesenchyme, while *Brd10* expression is undetectable. In the adult valve, *Dzip1* remains detectable at relatively low levels, whereas *Brd10* is re-expressed predominantly within endothelial cells. AV cushions and leaflets are outlined. BL indicates background staining caused by blood autofluorescence. Hoechst nuclear counterstain is shown in blue. AL, anterior leaflet; PL, posterior leaflet; AVC, atrioventricular cushion.

To test whether *Dzip1* and *Brd10* are co-expressed in the same cell type, RNAscope analyses was performed during cardiac development on embryonic day 13.5 (E13.5) and postnatal day 0, and in the adult (6 months old) (Figure 3). *Brd10* mRNA is expressed at E13.5 in the endothelium and mesenchyme of the developing atrioventricular (AV) cushions. Consistent with prior studies, *Dzip1* mRNA expression was also observed at this timepoint, primarily restricted to the mesenchyme [8,9,11]. Overlap of mRNA for *Dzip1* and *Brd10* demonstrated co-expression in most of the mesenchymal cells present within the AV cushions. *Dzip1* mRNA expression persisted in the mitral valve mesenchyme at P0 and at 6 months of age, although at relatively lower levels. In contrast, *Brd10* expression was not detected at P0 or in the adult mitral leaflets at 6 months.

**Figure 4:**
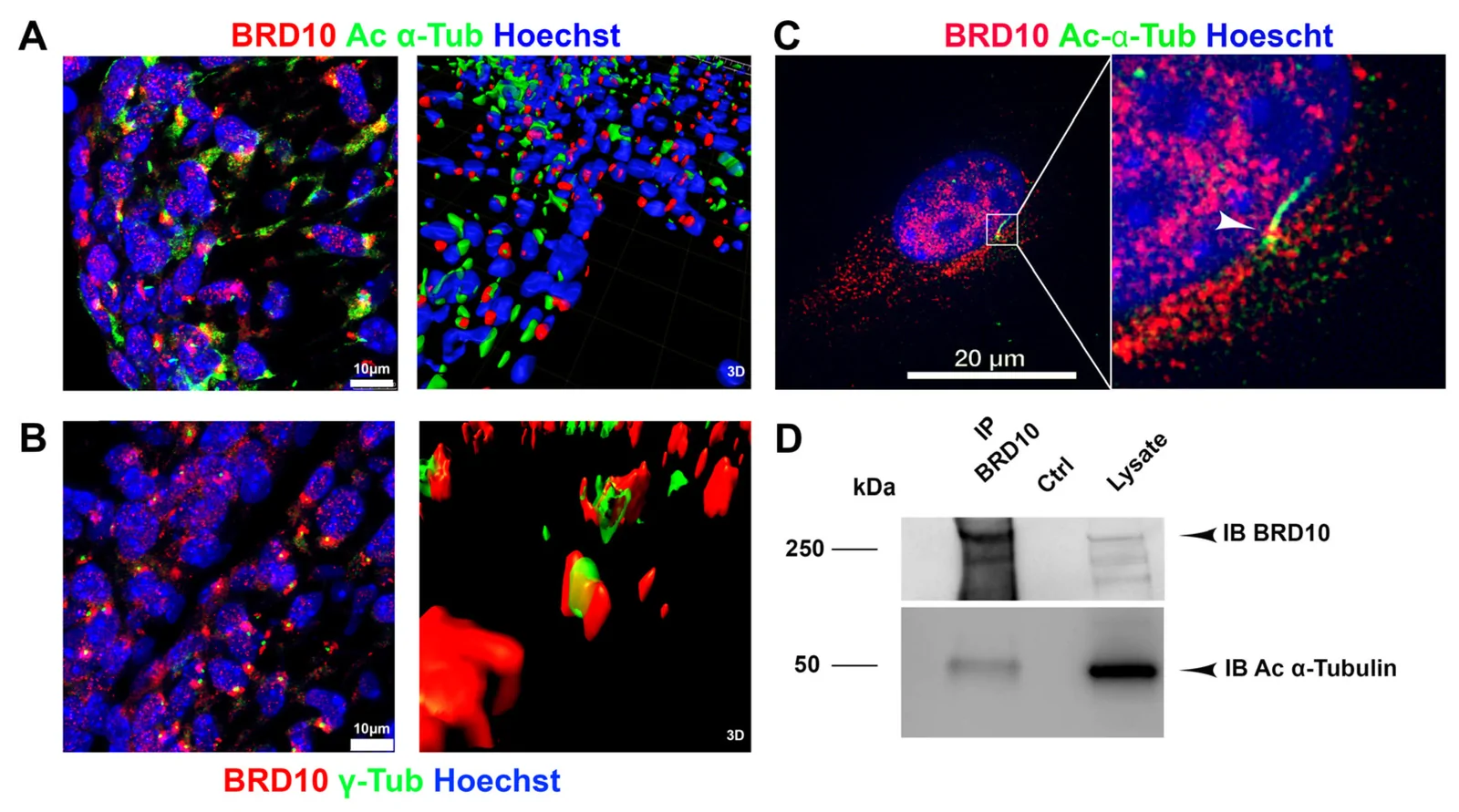
BRD10 localizes to the primary cilium and associates with acetylated α-tubulin. (**A**) IHC and 3D reconstructions of Z-stack staining of E13.5 AV cushions showing BRD10 (red) in close association with acetylated α-tubulin (green) at the primary cilium. (**B**) BRD10 localization relative to γ-tubulin demonstrates enrichment at the basal body/ciliary base in a pattern reminiscent of the ciliary necklace. Expression of BRD10 is also prominent in the nuclei. (**C**) ICC in serum-starved NIH3T3 cells shows BRD10 protein in the nucleus and near the ciliary base. The arrow indicates BRD10 expression across the base of the axoneme. (**D**) Co-IP demonstrates association between BRD10 and acetylated α-tubulin. Hoechst nuclear counterstain is shown in blue.

IHC analyses to examine protein localization of BRD10 demonstrated expression at the ciliary basal body as defined by γ-tubulin staining, with a characteristic necklace-like distribution around the ciliary base in E13.5 AV cushions and NIH3T3 cells (Figure 4A–C). Additional staining of BRD10 was observed within the nucleus in a punctate pattern at E13.5 (Figure 4A,B) and within the nuclei of cultured cells (Figure 4C). This localization pattern was consistent with our previous observations of DZIP1 at the same developmental timepoint [9,11]. Notably, BRD10 localization at the basal body overlapped with both acetylated α-tubulin and γ-tubulin. As bromodomain-containing proteins preferentially recognize acetylated lysine residues, we tested whether BRD10 could physically associate with acetylated α-tubulin, a major component of the ciliary axoneme. NIH3T3 cells were serum-starved to promote ciliogenesis and immunostained for BRD10 and acetylated α-tubulin, confirming their spatial association at the cilium (Figure 4C). Co-IP followed by Western blot analysis further demonstrated an interaction between BRD10 and acetylated α-tubulin in vitro (Figure 4D), supporting a potential role for BRD10 in associating with acetylated microtubule structures at the base of the primary cilium. We do note that, although BRD10 expression has not been well documented, the GTEx portal (https://gtexportal.org/home/gene/KIAA2026/ (accessed 2 October 2026) shows broad expression throughout most organs and tissues, indicating a potential ubiquitous or certainly widespread function of this protein.

**Figure 5:**
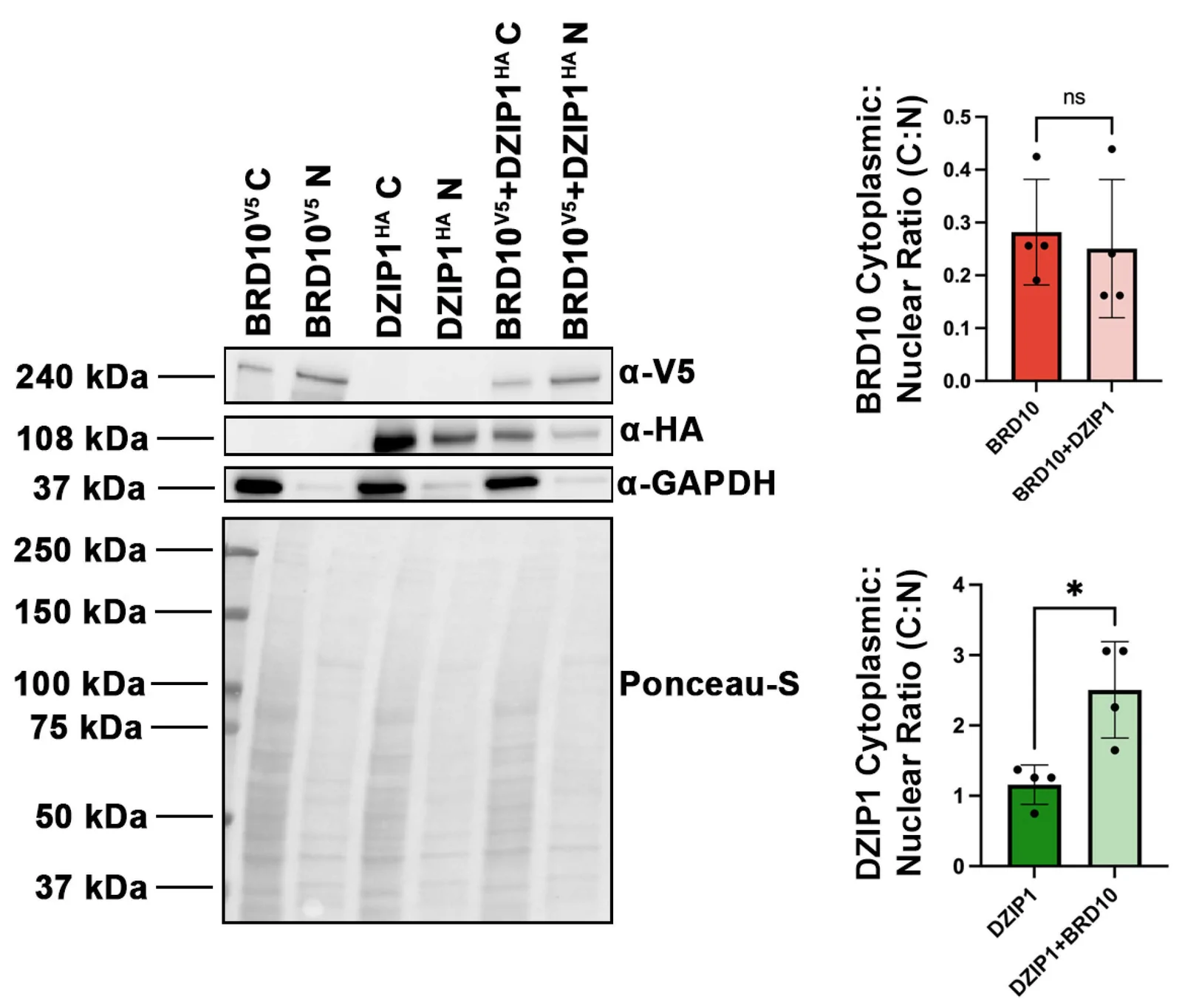
BRD10 alters the subcellular distribution of DZIP1. Nuclear and cytoplasmic fractionation of cells expressing BRD10 and/or DZIP1 was used to assess whether either protein influences the localization of the other. BRD10 was detected in both nuclear and cytoplasmic fractions and showed no significant change in its cytoplasmic-to-nuclear ratio following DZIP1 co-expression. In contrast, BRD10 co-expression significantly increased the cytoplasmic-to-nuclear ratio of DZIP1, driven primarily by a relative reduction in nuclear DZIP1. GAPDH was used as a cytoplasmic marker, and Ponceau S staining confirmed protein loading. Quantification is shown as cytoplasmic-to-nuclear (C:N) ratio; ns, not significant; * *p* < 0.05.

Given that an interaction between DZIP1 and BRD10 occurs within a putative nuclear localization sequence of BRD10, we hypothesized that DZIP1 may hide or hinder the transport of BRD10 into the nucleus and thereby control the subcellular localization of at least a subset of BRD10 protein at the base of the primary cilia. This was tested by generating bicistronic DZIP1/BRD10 constructs and testing them in vitro: 72 h post transfection, subcellular fractionation of cells was performed to separate nuclear vs. cytoplasmic fractions and tested on Western blot (Figure 5). BRD10 was detected in both compartments but showed greater relative abundance in the nuclear fraction, and alternate to our hypothesis, co-expression of DZIP1 did not significantly alter its cytoplasmic-to-nuclear distribution. In contrast, co-expression of BRD10 significantly shifted the distribution of DZIP1 toward the cytoplasmic compartment, reflected by an increased cytoplasmic ratio. This effect appeared to be driven primarily by a relative reduction in nuclear DZIP1 rather than an increase in absolute cytoplasmic abundance. Together, these findings suggest that, while DZIP1 does not affect BRD10 localization, BRD10 does influence the subcellular distribution of DZIP1.

## 4. Discussion

Mitral valve prolapse (MVP) is increasingly viewed as a disease in which developmental abnormalities can establish the substrate for progressive myxomatous degeneration later in life. Although the mature diseased valve is characterized by striking changes in ECM composition and leaflet architecture, genetic and experimental studies point to earlier defects in cell organization, signaling, mechanosensing, and tissue patterning [2,8,9,11,12,29,30,31,32,33,34,35,36,37,38,39,40,41]. This is supported by human genetics identifying many developmentally expressed genes shown to be causative of MVP in families and GWAS studies, including DCHS1, FLNA, DZIP1, and TNS1. The primary cilium appear to be particularly important in this context, especially since loss or mutations in many of these genes can alter the presence of cilia or their signaling roles. Our prior studies, and those of others, have also shown that disruption of ciliogenesis during valvulogenesis alters ECM organization and valve architecture, while loss-of-function variants in DZIP1 provide a direct genetic link between defective ciliary biology and human MVP.

The present study identifies BRD10 as a previously unrecognized DZIP1-interacting protein and suggests that this interaction may influence where DZIP1 is positioned within the cell. This may be more important than changes in total protein abundance. Primary cilia are highly compartmentalized organelles, and entry of proteins into the ciliary axoneme is tightly regulated at the ciliary base by transition fibers, the transition zone, and the ciliary necklace [42,43,44,45]. Thus, proteins that control the positioning or retention of DZIP1 could alter ciliary signaling even when DZIP1 itself is normally expressed. Several findings support such a model. BRD10 interacted with DZIP1 using independent biochemical approaches, localized near the basal body of the primary cilium, and was detected in overlapping proximity with γ-tubulin and acetylated α-tubulin. BRD10 also co-immunoprecipitated with acetylated α-tubulin. This interaction may be especially relevant because acetylated tubulin is highly enriched in stable ciliary microtubules [46,47]. Binding to acetylated tubulin may provide an anchor that stabilizes BRD10-containing protein complexes at the ciliary base or along the proximal axoneme. Such an interaction could help maintain DZIP1 and other associated proteins within a defined ciliary signaling compartment. Our fractionation studies are consistent with BRD10 influencing DZIP1 localization rather than the reverse. DZIP1 had little effect on the nuclear-to-cytoplasmic distribution of BRD10. In contrast, BRD10 expression shifted DZIP1 toward the non-nuclear compartment, largely through a reduction in nuclear DZIP1. Because the cytoplasmic fraction also contains centrosomal, basal-body, and ciliary-associated proteins, this finding raises the possibility that BRD10 promotes retention of DZIP1 within one or more of these compartments. Whether this reflects altered nuclear trafficking, stabilization at the ciliary base, or recruitment into a larger protein complex will require further study.

The predicted structure of BRD10 provides support for a scaffolding role. Our AlphaFold analyses suggest that BRD10 is a large protein with extensive regions of low structural confidence consistent with substantial intrinsic disorder. Proteins with large intrinsically disordered regions often participate in dynamic, multivalent interactions rather than functioning as rigid structural proteins [48]. This property could allow BRD10 to assemble or stabilize transient complexes involving DZIP1, microtubules, trafficking machinery, and signaling proteins. Its association with acetylated tubulin could then provide a means of anchoring these complexes to the ciliary microtubule network. These observations raise the possibility that BRD10 and DZIP1 participate in a gatekeeping mechanism at the base of the cilium. This does not imply that BRD10 is itself an established component of the transition zone or ciliary necklace. Rather, BRD10 may help organize protein complexes near this selective barrier and thereby influence which proteins gain access to, or are retained within, the ciliary axoneme. This interpretation is consistent with emerging functions of DZIP1 beyond ciliogenesis alone. DZIP1 interacts with GLI3 and can regulate its localization, while also associating with proteins involved in ciliary trafficking and assembly, including CEP164 and IFT88 [49]. In the developing mitral valve, DZIP1 has also been linked to β-catenin signaling, shuttling, and ECM remodeling [9,50].

Hedgehog signaling provides a particularly interesting setting in which such a mechanism could operate. Primary cilia are required for several Hedgehog signaling pathways, and DZIP1 has previously been linked to GLI3 trafficking [9]. In the developing mitral valve, however, the relevant pathway appears to be more specialized. Desert Hedgehog (DHH), rather than Sonic or Indian Hedgehog, is uniquely expressed within the developing valve and signals through cilia to regulate cytoskeletal organization and valve morphogenesis [8]. DHH-dependent signaling promotes Smoothened localization to the ciliary axoneme and activates a TIAM1–RAC1 pathway that regulates actin organization. Disruption of this pathway produces abnormal valve development followed by features of myxomatous degeneration [8]. This valve-specific use of Hedgehog signaling makes control of ciliary access particularly relevant. The biological consequence may depend not simply on whether a cilium is present but on whether selected receptors, signaling intermediates, or regulatory proteins are able to enter and remain within the axoneme. A BRD10–DZIP1 complex anchored through acetylated tubulin could help establish such a signaling platform. Disturbing that organization might therefore change the response to DHH or other developmental signals without completely eliminating the cilium. This type of partial signaling defect is attractive in the context of MVP, where relatively subtle developmental abnormalities can become amplified over years of postnatal mechanical loading.

Taken together, our findings support a model in which BRD10 helps organize DZIP1-containing complexes at the primary cilium. Its interaction with DZIP1, localization near the ciliary base, association with acetylated α-tubulin, effect on DZIP1 compartmentalization, and predicted largely disordered structure are consistent with a dynamic scaffolding function. We propose that binding to acetylated α-tubulin may help anchor these complexes at the ciliary base, where BRD10 and DZIP1 could influence the movement or retention of ciliary proteins entering and exiting the axoneme. In the developing valve, disruption of this spatial control could alter cilia-dependent signaling, cytoskeletal remodeling, and ECM organization, providing another mechanism through which an early developmental defect could ultimately contribute to MVP.

## 5. Limitations

Some limitations should be noted. First, much of the work was performed using overexpression systems, which provide a useful platform for defining protein–protein interactions and subcellular distribution but do not fully recapitulate the cellular environment of developing mitral valve cells. Second, although BRD10 localized near the ciliary basal body and associated with acetylated α-tubulin, these experiments do not establish that BRD10 is required for ciliogenesis, ciliary stability, or ciliary signaling. Functional studies involving BRD10 loss- and gain-of-function will be required to determine whether altered BRD10 activity changes ciliary number, length, structure, signaling, or DZIP1 recruitment to the cilium. Additionally, specific mutation of the putative NLS/DZIP1 interaction motif may be particularly revealing of the molecular interactions occurring at the ciliary base or nuclear shuttling. Third, the developmental expression data demonstrate co-expression of *Brd10* and *Dzip1* mRNA in the embryonic valve, but co-expression alone does not establish that the proteins interact in vivo or that BRD10 is required for valve morphogenesis. Genetic studies of *Brd10* in animal models will be needed to determine whether its disruption produces abnormalities in ciliogenesis, ECM organization, leaflet architecture, or postnatal valve function. To date, a loss-of-function *Brd10* mouse has yet to be constructed or analyzed. Fourth, the localization of DZIP1 and BRD10 within the nucleus and control of DZIP1 nuclear localization by BRD10 is of potential relevance and may indicate that these proteins can interact within the nucleus to more broadly affect transcriptional programs at the primary cilium independent of their roles. Future studies examining BRD10/DZIP1 localization at single-cell resolution will be important for confirming this redistribution and determining whether DZIP1 is specifically retained at the basal body or in other cytoplasmic compartments. Finally, while the relationship between BRD10 and DZIP1 provides a compelling connection to a known MVP pathway, the present study does not demonstrate that BRD10 variants cause human MVP. Genetic analysis of MVP cohorts and functional evaluation of human BRD10 variants will be necessary before BRD10 can be implicated directly in human disease.

## 6. Conclusions

Taken together, this study identifies BRD10 as a previously unrecognized DZIP1-interacting protein and places it within the ciliary machinery relevant to mitral valve development. *Brd10* is co-expressed with *Dzip1* during valvulogenesis, localizes to the ciliary base, associates with acetylated α-tubulin, and alters the subcellular distribution of DZIP1. BRD10’s predicted disordered structure is also consistent with a potential scaffolding role that could help stabilize DZIP1-containing complexes at the cilium. These findings support a model in which BRD10 contributes to the spatial organization of ciliary signaling rather than simply to cilium formation itself. Because disruption of cilia-dependent signaling can alter cytoskeletal organization, ECM patterning, and valve architecture, this BRD10–DZIP1 interaction provides a new mechanistic link between ciliary protein trafficking and the developmental origins of MVP.

## Data Availability

Research data can be shared through the corresponding author upon reasonable request.

## Abbreviations

AV	Atrioventricular
BRD10	Bromodomain-containing protein 10
BSA	Bovine serum albumin
CAID	Critical Assessment of Intrinsic Disorder
cDNA	Complementary DNA
Co-IP	Co-immunoprecipitation
DBD	DNA-binding domain
DHH	Desert Hedgehog
DMEM	Dulbecco’s modified Eagle medium
DZIP1	DAZ-interacting zinc finger protein 1
ECM	Extracellular matrix
EndoMT	Endothelial-to-mesenchymal transition
GFP	Green fluorescent protein
GAPDH	Glyceraldehyde-3-phosphate dehydrogenase
HA	Hemagglutinin
HCF	Human cardiac fibroblast
HRP	Horseradish peroxidase
IHC	Immunohistochemistry
NLS	Nuclear localization signal
MVP	Mitral valve prolapse
PBS	Phosphate-buffered saline
P2A	Porcine teschovirus-1 2A peptide
SDS-PAGE	Sodium dodecyl sulfate–polyacrylamide gel electrophoresis

## References

[1] de Vlaming A., Sauls K., Hajdu Z., Visconti R.P., Mehesz A.N., Levine R.A., Slaugenhaupt S.A., Hagege A., Chester A.H., Markwald R.R. (2012). Atrioventricular valve development: New perspectives on an old theme. Differentiation.

[2] Delling F.N., Noseworthy P.A., Adams D.H., Basso C., Borger M., Bouatia-Naji N., Elmariah S., Evans F., Gerstenfeld E., Hung J. (2022). Research Opportunities in the Treatment of Mitral Valve Prolapse: JACC Expert Panel. J. Am. Coll. Cardiol..

[3] Levine R.A., Hagege A.A., Judge D.P., Padala M., Dal-Bianco J.P., Aikawa E., Beaudoin J., Bischoff J., Bouatia-Naji N., Bruneval P. (2015). Mitral valve disease—Morphology and mechanisms. Nat. Rev. Cardiol..

[4] Christensen S.T., Pedersen S.F., Satir P., Veland I.R., Schneider L. (2008). The primary cilium coordinates signaling pathways in cell cycle control and migration during development and tissue repair. Curr. Top. Dev. Biol..

[5] Hsiao Y.C., Tuz K., Ferland R.J. (2012). Trafficking in and to the primary cilium. Cilia.

[6] Veland I.R., Awan A., Pedersen L.B., Yoder B.K., Christensen S.T. (2009). Primary cilia and signaling pathways in mammalian development, health and disease. Nephron Physiol..

[7] Fulmer D., Toomer K., Guo L., Moore K., Glover J., Moore R., Stairley R., Lobo G., Zuo X., Dang Y. (2019). Defects in the Exocyst-Cilia Machinery Cause Bicuspid Aortic Valve Disease and Aortic Stenosis. Circulation.

[8] Fulmer D., Toomer K.A., Glover J., Guo L., Moore K., Moore R., Stairley R., Gensemer C., Abrol S., Rumph M.K. (2020). Desert hedgehog-primary cilia cross talk shapes mitral valve tissue by organizing smooth muscle actin. Dev. Biol..

[9] Guo L., Beck T., Fulmer D., Ramos-Ortiz S., Glover J., Wang C., Moore K., Gensemer C., Morningstar J., Moore R. (2021). DZIP1 regulates mammalian cardiac valve development through a Cby1-beta-catenin mechanism. Dev. Dyn..

[10] Toomer K.A., Fulmer D., Guo L., Drohan A., Peterson N., Swanson P., Brooks B., Mukherjee R., Body S., Lipschutz J.H. (2017). A role for primary cilia in aortic valve development and disease. Dev. Dyn..

[11] Toomer K.A., Yu M., Fulmer D., Guo L., Moore K.S., Moore R., Drayton K.D., Glover J., Peterson N., Ramos-Ortiz S. (2019). Primary cilia defects causing mitral valve prolapse. Sci. Transl. Med..

[12] Moore K.S., Moore R., Fulmer D.B., Guo L., Gensemer C., Stairley R., Glover J., Beck T.C., Morningstar J.E., Biggs R. (2022). DCHS1, Lix1L, and the Septin Cytoskeleton: Molecular and Developmental Etiology of Mitral Valve Prolapse. J. Cardiovasc. Dev. Dis..

[13] Chen P., Zuo N., Wu C., Ma J., Li Y., Gu J., Li W., Liu S. (2021). MECOM promotes supporting cell proliferation and differentiation in cochlea. J. Otol..

[14] Leszczyński P., Śmiech M., Teeli A.S., Haque E., Viger R., Ogawa H., Pierzchała M., Taniguchi H. (2020). Deletion of the Prdm3 Gene Causes a Neuronal Differentiation Deficiency in P19 Cells. Int. J. Mol. Sci..

[15] Pluteanu F., Glaser D., Massing F., Schulte J.S., Kirchhefer U. (2024). Loss of protein phosphatase 2A regulatory subunit PPP2R5A is associated with increased incidence of stress-induced proarrhythmia. Front. Cardiovasc. Med..

[16] Mao Z., Liu C., Lin X., Sun B., Su C. (2018). PPP2R5A: A multirole protein phosphatase subunit in regulating cancer development. Cancer Lett..

[17] Jin Z., Mei W., Strack S., Jia J., Yang J. (2011). The antagonistic action of B56-containing protein phosphatase 2As and casein kinase 2 controls the phosphorylation and Gli turnover function of Daz interacting protein 1. J. Biol. Chem..

[18] Mendez M.G., Kojima S.-I., Goldman R.D. (2010). Vimentin induces changes in cell shape, motility, and adhesion during the epithelial to mesenchymal transition. FASEB J..

[19] Jiu Y., Lehtimäki J., Tojkander S., Cheng F., Jäälinoja H., Liu X., Varjosalo M., Eriksson J.E., Lappalainen P. (2015). Bidirectional Interplay between Vimentin Intermediate Filaments and Contractile Actin Stress Fibers. Cell Rep..

[20] Yan W., Deng Y., Zhang Y., Luo J., Lu D., Wan Q., Mao L., Chen Y. (2019). DZIP1 Promotes Proliferation, Migration, and Invasion of Oral Squamous Carcinoma Through the GLI1/3 Pathway. Transl. Oncol..

[21] Li F.-Q., Chen X., Fisher C., Siller S.S., Zelikman K., Kuriyama R., Takemaru K.-I. (2016). BAR Domain-Containing FAM92 Proteins Interact with Chibby1 To Facilitate Ciliogenesis. Mol. Cell. Biol..

[22] Zhang H., Liu Y., Zhang K., Hong Z., Liu Z., Liu Z., Li G., Xu Y., Pi J., Fu J. (2024). Understanding the Transcription Factor NFE2L1/NRF1 from the Perspective of Hallmarks of Cancer. Antioxidants.

[23] Filippakopoulos P., Picaud S., Mangos M., Keates T., Lambert J.-P., Barsyte-Lovejoy D., Felletar I., Volkmer R., Müller S., Pawson T. (2012). Histone Recognition and Large-Scale Structural Analysis of the Human Bromodomain Family. Cell.

[24] Jacobson R.H., Ladurner A.G., King D.S., Tjian R. (2000). Structure and Function of a Human TAFII250 Double Bromodomain Module. Science.

[25] Kasper L.H., Fukuyama T., Biesen M.A., Boussouar F., Tong C., Pauw A.d., Murray P.J., Deursen J.M.A.v., Brindle P.K. (2006). Conditional Knockout Mice Reveal Distinct Functions for the Global Transcriptional Coactivators CBP and p300 in T-Cell Development. Mol. Cell. Biol..

[26] Lamonica J.M., Deng W., Kadauke S., Campbell A.E., Gamsjaeger R., Wang H., Cheng Y., Billin A.N., Hardison R.C., Mackay J.P. (2011). Bromodomain protein Brd3 associates with acetylated GATA1 to promote its chromatin occupancy at erythroid target genes. Proc. Natl. Acad. Sci. USA.

[27] Abramson J., Adler J., Dunger J., Evans R., Green T., Pritzel A., Ronneberger O., Willmore L., Ballard A.J., Bambrick J. (2024). Accurate structure prediction of biomolecular interactions with AlphaFold 3. Nature.

[28] Jumper J., Evans R., Pritzel A., Green T., Figurnov M., Ronneberger O., Tunyasuvunakool K., Bates R., Zidek A., Potapenko A. (2021). Applying and improving AlphaFold at CASP14. Proteins.

[29] Clemenceau A., Berube J.C., Belanger P., Gaudreault N., Lamontagne M., Toubal O., Clavel M.A., Capoulade R., Mathieu P., Pibarot P. (2018). Deleterious variants in DCHS1 are prevalent in sporadic cases of mitral valve prolapse. Mol. Genet. Genom. Med..

[30] Delling F.N., Vasan R.S. (2014). Epidemiology and pathophysiology of mitral valve prolapse: New insights into disease progression, genetics, and molecular basis. Circulation.

[31] Devereux R.B., Brown W.T., Kramer-Fox R., Sachs I. (1982). Inheritance of mitral valve prolapse: Effect of age and sex on gene expression. Ann. Intern. Med..

[32] Dieterlen M.T., Klaeske K., Spampinato R., Marin-Cuartas M., Wiesner K., Morningstar J., Norris R.A., Melnitchouk S., Levine R.A., van Kampen A. (2023). Histopathological insights into mitral valve prolapse-induced fibrosis. Front. Cardiovasc. Med..

[33] Dina C., Bouatia-Naji N., Tucker N., Delling F.N., Toomer K., Durst R., Perrocheau M., Fernandez-Friera L., Solis J., PROMESA investigators (2015). Genetic association analyses highlight biological pathways underlying mitral valve prolapse. Nat. Genet..

[34] Disse S., Abergel E., Berrebi A., Houot A.M., Le Heuzey J.Y., Diebold B., Guize L., Carpentier A., Corvol P., Jeunemaitre X. (1999). Mapping of a first locus for autosomal dominant myxomatous mitral-valve prolapse to chromosome 16p11.2-p12.1. Am. J. Hum. Genet..

[35] Durst R., Sauls K., Peal D.S., deVlaming A., Toomer K., Leyne M., Salani M., Talkowski M.E., Brand H., Perrocheau M. (2015). Mutations in DCHS1 cause mitral valve prolapse. Nature.

[36] Duval D., Labbe P., Bureau L., Le Tourneau T., Norris R.A., Markwald R.R., Levine R., Schott J.J., Merot J. (2015). MVP-Associated Filamin A Mutations Affect FlnA-PTPN12 (PTP-PEST) Interactions. J. Cardiovasc. Dev. Dis..

[37] Grau J.B., Pirelli L., Yu P.J., Galloway A.C., Ostrer H. (2007). The genetics of mitral valve prolapse. Clin. Genet..

[38] Morningstar J.E., Nieman A., Wang C., Beck T., Harvey A., Norris R.A. (2021). Mitral Valve Prolapse and Its Motley Crew-Syndromic Prevalence, Pathophysiology, and Progression of a Common Heart Condition. J. Am. Heart Assoc..

[39] Norris R.A., Moreno-Rodriguez R., Wessels A., Merot J., Bruneval P., Chester A.H., Yacoub M.H., Hagege A., Slaugenhaupt S.A., Aikawa E. (2010). Expression of the familial cardiac valvular dystrophy gene, filamin-A, during heart morphogenesis. Dev. Dyn..

[40] Roselli C., Yu M., Nauffal V., Georges A., Yang Q., Love K., Weng L.C., Delling F.N., Maurya S.R., Schrolkamp M. (2022). Genome-wide association study reveals novel genetic loci: A new polygenic risk score for mitral valve prolapse. Eur. Heart J..

[41] Yu M., Kyryachenko S., Debette S., Amouyel P., Schott J.J., Le Tourneau T., Dina C., Norris R.A., Hagege A.A., Jeunemaitre X. (2021). Genome-Wide Association Meta-Analysis Supports Genes Involved in Valve and Cardiac Development to Associate with Mitral Valve Prolapse. Circ. Genom. Precis. Med..

[42] Hu Q., Nelson W.J. (2011). Ciliary diffusion barrier: The gatekeeper for the primary cilium compartment. Cytoskeleton.

[43] Reiter J.F., Blacque O.E., Leroux M.R. (2012). The base of the cilium: Roles for transition fibres and the transition zone in ciliary formation, maintenance and compartmentalization. EMBO Rep..

[44] Takao D., Verhey K.J. (2016). Gated entry into the ciliary compartment. Cell. Mol. Life Sci..

[45] Garcia-Gonzalo F.R., Reiter J.F. (2017). Open Sesame: How Transition Fibers and the Transition Zone Control Ciliary Composition. Cold Spring Harb. Perspect. Biol..

[46] Guichard P., Laporte M.H., Hamel V. (2023). The centriolar tubulin code. Semin. Cell Dev. Biol..

[47] Nekooki-Machida Y., Hagiwara H. (2020). Role of tubulin acetylation in cellular functions and diseases. Med. Mol. Morphol..

[48] Weng J., Wang W. (2020). Dynamic multivalent interactions of intrinsically disordered proteins. Curr. Opin. Struct. Biol..

[49] Wang C., Low W.C., Liu A., Wang B. (2013). Centrosomal protein DZIP1 regulates Hedgehog signaling by promoting cytoplasmic retention of transcription factor GLI3 and affecting ciliogenesis. J. Biol. Chem..

[50] Zhang B., Zhang T., Wang G., Wang G., Chi W., Jiang Q., Zhang C. (2015). GSK3beta-*Dzip1*-Rab8 cascade regulates ciliogenesis after mitosis. PLoS Biol..

